# Acupuncture Treatment Modulates the Connectivity of Key Regions of the Descending Pain Modulation and Reward Systems in Patients with Chronic Low Back Pain

**DOI:** 10.3390/jcm9061719

**Published:** 2020-06-03

**Authors:** Siyi Yu, Ana Ortiz, Randy L. Gollub, Georgia Wilson, Jessica Gerber, Joel Park, Yiting Huang, Wei Shen, Suk-Tak Chan, Ajay D. Wasan, Robert R. Edwards, Vitaly Napadow, Ted J. Kaptchuk, Bruce Rosen, Jian Kong

**Affiliations:** 1 Department of Psychiatry, Massachusetts General Hospital, Harvard Medical School, Charlestown, MA 02129, USA; cdutcmysy@gmail.com (S.Y.); ana.ortiz@utsouthwestern.edu (A.O.); RGOLLUB@PARTNERS.ORG (R.L.G.); GJWILSON@mgh.harvard.edu (G.W.); JPARK51@partners.org (J.P.); yiting.h@hotmail.com (Y.H.); hy0204033@hainmc.edu.cn (W.S.); 2 Department of Radiology, Martinos Center for Biomedical Imaging, Massachusetts General Hospital, Harvard Medical School, Charlestown, MA 02129, USA; JGERBER2@mgh.harvard.edu (J.G.); phoebe@nmr.mgh.harvard.edu (S.-T.C.); vitaly@mgh.harvard.edu (V.N.); bruce@nmr.mgh.harvard.edu (B.R.); 3 Department of Anesthesiology, Center for Pain Research, University of Pittsburgh, Pittsburgh, PA 15206, USA; wasanad@upmc.edu; 4 Department of Anesthesiology, Perioperative and Pain Medicine, Brigham and Women’s Hospital, Harvard Medical School, Boston, MA 02467, USA; rredwards@bwh.harvard.edu; 5 Beth Israel Deaconess Medical Center, Harvard Medical School, Boston, MA 02215, USA; ted_kaptchuk@hms.harvard.edu

**Keywords:** acupuncture, chronic low back pain, descending pain modulation system, reward network, functional connectivity, PAG, VTA

## Abstract

Chronic low back pain (cLBP) is a common disorder with unsatisfactory treatment options. Acupuncture has emerged as a promising method for treating cLBP. However, the mechanism underlying acupuncture remains unclear. In this study, we investigated the modulation effects of acupuncture on resting state functional connectivity (rsFC) of the periaqueductal gray (PAG) and ventral tegmental area (VTA) in patients with cLBP. Seventy-nine cLBP patients were recruited and assigned to four weeks of real or sham acupuncture. Resting state functional magnetic resonance imaging data were collected before the first and after the last treatment. Fifty patients completed the study. We found remission of pain bothersomeness in all treatment groups after four weeks, with greater pain relief after real acupuncture compared to sham acupuncture. We also found that real acupuncture can increase VTA/PAG rsFC with the amygdala, and the increased rsFC was associated with decreased pain bothersomeness scores. Baseline PAG-amygdala rsFC could predict four-week treatment response. Our results suggest that acupuncture may simultaneously modulate the rsFC of key regions in the descending pain modulation (PAG) and reward systems (VTA), and the amygdala may be a key node linking the two systems to produce antinociceptive effects. Our findings highlight the potential of acupuncture for chronic low back pain management.

## 1. Introduction

Low back pain (LBP) is a common disorder with an estimated lifetime prevalence of 70% to 85% [1]. Current treatments for chronic low back pain (cLBP) are often unsatisfactory [2]. Despite the controversies regarding the use of opioids to treat chronic low back pain [3], they remain the most commonly prescribed class of drugs for the disorder. In the past two decades, opioid prescriptions have increased worldwide [4,5], especially in the United States, where opioid sales quadrupled from 1999 to 2010 [6]. Furthermore, an increase in opioid misuse and complications has emerged as a serious substance abuse crisis, highlighting the need for nonopioid treatments for chronic pain.

Recently, accumulating evidence has endorsed the potential of acupuncture treatment for chronic pain, including chronic low back pain [7,8]. Based on these findings, a guideline [9] from the American College of Physicians has strongly recommended acupuncture as a treatment option for cLBP. However, the underlying mechanism of acupuncture treatment remains unclear, which has hindered the development and incorporation of this promising treatment into mainstream medicine. 

Studies suggest that the opioidergic descending pain modulation system (DPMS) plays an important role in acupuncture treatment of chronic pain [10,11,12,13,14,15]. In addition, literature suggests that cLBP is associated with neural plasticity changes in the DPMS and limbic system, including the prefrontal cortex (PFC), anterior cingulate cortex (ACC), hippocampus, amygdala, periaqueductal gray (PAG), and rostroventromedial medulla (RVM) [16,17,18,19,20]. Studies also indicate that the PAG, an area enriched with opioidergic neurons [21], may act as a critical hub in the neuroaxis of the DPMS [13]. In an earlier study, we found increased resting state functional connectivity (rsFC) between the PAG and the medial prefrontal cortex (mPFC)/ACC in cLBP patients compared to healthy controls (HCs) [22].

Pain is fundamentally an unpleasant experience. The aversiveness of pain, as well as the reward from pain relief, is encoded by the brain reward/motivational system [23]. Literature suggests that the dopamine reward system may also be disrupted in chronic pain patients. Studies have found that chronic pain is a hypodopaminergic state [24,25,26]. The ventral tegmental area (VTA), a key structure in the midbrain, sends dopaminergic neural projections to the ventral striatum (i.e., nucleus accumbens), limbic system (e.g., hippocampus and amygdala), and prefrontal cortex (e.g., mPFC, ACC, and OFC), forming the mesocorticolimbic system [27,28,29,30,31]. In a recent study, we found that chronic low back pain was associated with VTA functional connectivity alterations [32], and preclinical studies have suggested that targeting reward/motivation circuits may promote recovery from chronic pain [23].

Several lines of evidence suggest that the dopamine reward and opioid descending pain modulation systems are neuroanatomically related and interact in complex ways to modulate pain processing [24]. Studies suggest that pain and pain relief may be associated with activation of opioidergic and dopaminergic cortico-limbic circuits [33]. For instance, studies have shown that dopamine, a key neurotransmitter of reward, is involved in pain modulation [34], and opioids are central to endogenous pain inhibition and are also involved in reward processing [24]. In a recent study, we found that exercise can simultaneously modulate functional connectivity of the descending pain modulation pathway and the reward/motivation system to relieve knee osteoarthritis pain [35].

Thus, in this study, we simultaneously investigated the modulation effects of acupuncture treatment on the descending pain modulation system (using the PAG as “seed”, an a priori selected region of interest for voxelwise functional connectivity analysis) and on the reward system (using the VTA as seed) in cLBP patients. Resting state fMRI data was collected at baseline and after four weeks of either real or sham acupuncture treatment in a single-blinded trial. Previous studies have suggested that expectancy/context may modulate the acupuncture treatment response [36,37,38,39,40], and we also attempted to evaluate the context effect using a context manipulation model [41]. We hypothesized that acupuncture would significantly modulate rsFC of the DPMS and reward network and that expectancy would further modulate acupuncture treatment effects.

## 2. Materials and Methods

Experiments were conducted with approval from the Massachusetts General Hospital Institutional Review Board, and the written, informed consent of each participant was obtained prior to study procedures. Participants were debriefed about the true nature of the study after completion of all procedures. This study was registered on ClinicalTrials.gov (NCT01595451). 

It should be noted that part of the MRI data set (baseline resting state fMRI data) was used in a recent study [42] to explore the feasibility of predicting treatment response using a data-driven brain imaging method (independent component analysis). This study applied a hypothesis-driven method (seed-based functional connectivity) to investigate how four weeks of real and sham acupuncture can modulate the descending pain modulation system and reward/motivation network by comparing pre- and post-treatment PAG and VTA connectivity differences across different groups, which has not been previously published. 

## 3. Participants

Patients with chronic low back pain (n = 79) were enrolled in the study if they met all inclusion criteria and no exclusion criteria.

Inclusion criteria were: (1) 18–60 years old, (2) presence of cLBP for a duration of at least six months or longer [43], (3) pain intensity average of at least a 4 on the 0–10 visual analog scale (VAS) during the screening, and (4) having not participated in acupuncture treatment for at least one year.

Exclusion criteria were: (1) specific causes of back pain (e.g., cancer, fractures, spinal stenosis, infections), (2) complicated back problems (e.g., prior back surgery, medicolegal issues), (3) major systemic diseases or history of head injury or coma, (4) possible contraindications for acupuncture (e.g., coagulation disorders, pregnancy) and conditions that might confound longitudinal effects or interpretation of results (e.g., severe fibromyalgia, rheumatoid arthritis), (5) presence of any contraindications to MRI scanning (for example: cardiac pacemaker, metal implants, claustrophobia, pregnancy, inability to lie still in fMRI scanner), and (6) history of substance abuse or dependence based on self-report. 

## 4. Experimental Procedures

**Figure 1:**
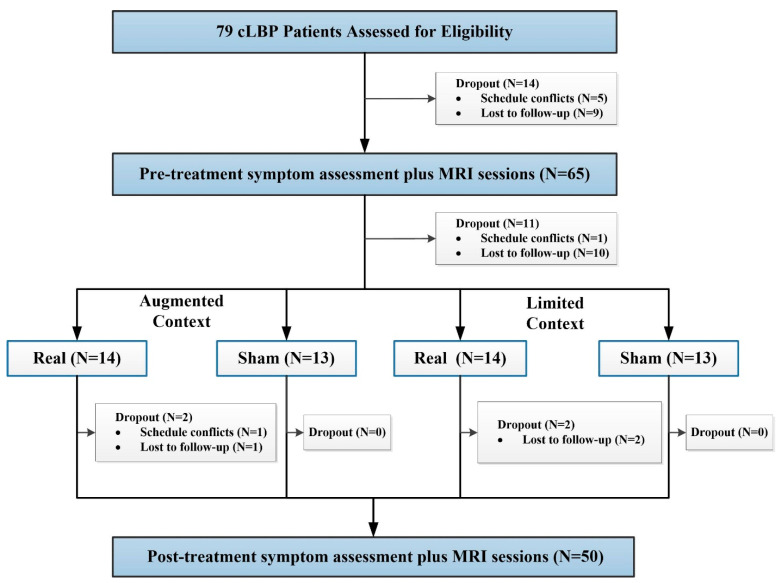
Procedures and data used for the study.

Using a permuted block randomization, patients were randomized into one of four groups: “augmented context” real acupuncture, “limited context” real acupuncture, “augmented context” sham acupuncture, or “limited context” sham acupuncture. All patients and study staff were blinded to the treatment groups. Only the acupuncturists, who had to know whether to deliver real or sham treatment, were not blinded. Details of the study design can be found in Figure 1. 

The first treatment session consisted of a context manipulation and an acupuncture administration. The acupuncturist was informed whether the random context assignment was “augmented” or “limited” right before the subject entered the treatment room. Immediately before needling, the acupuncturist was informed whether to use real or sham needles according to a second nested randomization schema. 

## 5. Acupuncture Treatment

All subjects received six identical manual acupuncture treatments over about four weeks according to the following schedule: two times per week for the first two weeks and one time per week for the last two weeks. Each real or sham treatment lasted for about 25 min and was performed by a licensed MGH acupuncturist. Additional stimulation was applied to elicit *deqi* by twirling the needles at 10 min and again just prior to needle removal.

For the real acupuncture group, we employed a modified standardized acupuncture protocol based on cLBP clinical trials [44]. We employed seven real acupoints considered by experts to be effective for chronic low back pain [44]: *Yaoyangguan* (GV3), bilateral *Shenshu* (BL23), bilateral *Weizhong* (BL40), bilateral *Taixi* (KI3), and 1-3 *ashi* points bilaterally on the lower back and legs (Table S1). This treatment protocol is considered effective by experts on chronic low back pain [44].

Sham acupuncture was applied at 12 sham acupuncture points (Table S1) using a Streitberger placebo acupuncture needle. Nonacupoints were located based on (1) relative closeness to the real points selected and (2) convenience of administration. Instead of penetrating the skin, the point of the Streitberger needle retracts up the handle shaft when the acupuncturist presses it against the skin. This sham device has been validated by studies showing that subjects are unable to distinguish between real and sham needling [45,46,47]. 

## 6. High- and Low-Context Manipulation 

Subjects randomly assigned to the “augmented” context experienced a structured interaction with the acupuncturist using a method applied in our previous study [41]. The acupuncturist’s interaction with the subject was structured with respect to both content (conversations) and style (five primary behaviors). The topics of discussion included questions concerning (1) LBP symptoms; (2) other medical symptoms; (3) psychosocial history, Chinese medicine intake, and how cLBP has affected the patient’s relationships and lifestyle; and (4) how the patient understands the “cause” and “meaning” of his or her condition. The acupuncturist incorporated four primary behaviors including: (1) exuding a warm, friendly manner; (2) active listening (such as repeating patient’s words, asking for clarifications); (3) empathy (such as saying “I can understand how difficult cLBP must be for you”); (4) 20 s of thoughtful silence while taking the patient’s pulse or pondering the treatment plan; and (5) communication of confidence and positive expectation. The subject also received physical contact from the acupuncturist during the Chinese medicine intake. The acupuncturist had a checklist to ensure that all key points were covered.

Meanwhile, in the “limited” context group, the acupuncturist merely read study information to the patient and aimed to “converse with patients as little as possible.” The details of the manipulation of context can be found in Supplementary Materials. 

We used the expectations for relief scale, a 0–10 scale (with 0 indicating a very negative expectation of “does not work at all” and 10 indicating a very positive expectation of “complete pain relief”), to measure the expectation of patients for acupuncture treatment at baseline, after Session 1, Session 4, and Session 6. This method has been used in our previous study [48,49].

## 7. Clinical Outcomes and Data Analysis

In this study, we used a bothersomeness scale to measure pain severity and used it as our primary clinical endpoint. We chose the bothersomeness scale based on a previous large clinical trial on acupuncture treatment of chronic LBP [50]. The scale measures how bothersome a patient’s LBP has been in the previous week, with responses varying on the VAS scale (0–10) from “not at all bothersome” (0) to “extremely bothersome” (10). This measure demonstrates substantial construct validity [51,52,53]. The Beck Depression Inventory was applied to assess the mental state of participants.

Clinical outcome analysis was performed using SPSS 22.0 Software (SPSS Inc., Chicago, IL, USA). Two sample *t*-tests and a chi-squared test were applied to compare the baseline characteristics of the subjects between groups. We first used a paired *t*-test to compare the pre- and post-treatment pain bothersomeness differences across each treatment group separately. We then applied a two-sample *t*-test to compare the pre- and post-treatment pain bothersomeness score change differences between (1) the real (augmented and limited context) and sham (augmented and limited context) acupuncture groups, and (2) the augmented context (real and sham acupuncture) and limited context (real and sham acupuncture) groups. Two meta-analyses on chronic pain found that acupuncture has a clinically relevant effect on chronic pain, and true acupuncture was significantly superior to sham control [7,8]. Based on these meta-analysis results, clinical practice guidelines [9], and our hypothesis, we tested (1) if real acupuncture can produce greater clinical improvement (pain bothersomeness score reduction) than sham acupuncture; and (2) if augmented context acupuncture can produce greater pain reduction than low context acupuncture. Thus, we applied a one-tailed hypothesis test in two sample *t*-tests when we compared real vs. sham acupuncture and high context vs. low context. 

## 8. fMRI Data Acquisition and Data Analysis

The MRI scans were performed before (baseline) and after six real or sham acupuncture treatments. All fMRI data was acquired using a 32-channel radio-frequency head coil in a 3T Siemens scanner at the Massachusetts General Hospital Martinos Center for Biomedical Imaging. A whole-brain gradient-echo echo-planar-imaging sequence was used for functional scanning with a repetition time of 3000 ms (30 ms echo time, 44 3.0 mm-thick slices, 2.6 × 2.6 mm in-plane resolution). A high-resolution, T1-weighted structural image (1 mm^3^ isotropic voxel MPRAGE) was acquired after functional imaging. During the resting-state fMRI, subjects were asked to keep their eyes open and to blink normally while looking at a darkened screen for approximately six minutes.

Data and calculations of functional connectivity were all preprocessed using the CONN toolbox version 17.f (http://www.nitrc.org/projects/conn) in MATLAB [54]. We used the default preprocessing pipeline for seed-to-voxel rsFC analysis. The specific steps were as follows: slice timing correction, head motion correction, skull-stripping using BET, co-registration of the anatomical image to the mean functional image, segmentation of the anatomical gray matter, white matter, and cerebrospinal fluid, normalization to MNI152 standard template, and smoothing with a 6-mm Gaussian kernel [54]. Band-pass filtering was performed with a frequency window of 0.01–0.08 Hz. 

To eliminate head motion and artifacts, we identified outlier time points in the motion parameters and global signal intensity using ART (http://www.nitrc.org/projects/artifact_detect). For each subject, we treated images (time points) as outliers if composite movement from a preceding image exceeded 0.5 mm, or if the global mean intensity was greater than three standard deviations from the mean image intensity for the entire resting scan. Outliers were included as regressors in the first level general linear model along with motion parameters. In addition, to investigate the effect of head motion during the resting state scans, mean framewise displacement [55] was calculated for each participant.

Based on previous studies, we used the bilateral ventrolateral PAG with a 3 mm-radius sphere (MNI coordinates *x* = ± 4, *y* = −26, *z* = −14) [56,57], as well as the bilateral VTA with a 4 mm-radius sphere (MNI coordinates *x* = −4, *y* = −15, *z* = −9; *x* = 5, *y* = −14, *z* = −8) [58], as regions of interest (ROIs) using a seed-to-voxel approach. The coordinates of the PAG seed in this study are from our previous study [57], in which we observed ventrolateral PAG activation to heat pain during task-based fMRI analyses. Following this early study, this PAG seed has been applied by our group and others in different chronic diseases, including menstrual pain [59], migraine [60], chronic neck and shoulder pain [61], and low back pain [22]. Therefore, we believe the PAG coordinates are applicable across different pain conditions/populations. Additionally, these coordinates are part of the ventrolateral PAG, which is believed to be important for opioid antinociception [62]. Similarly, the coordinates of the bilateral VTA were derived from a task fMRI study that showed increased activation in the VTA to reward-predicting cues [58]. 

In the first-level analysis, we produced a correlation map for each subject by extracting the blood oxygen level dependent time course from the bilateral PAG and VTA seeds separately and computing Pearson’s correlation coefficients between the time course in the PAG/VTA and every voxel of the whole brain. Seed-to-voxel between-group connectivity analyses were performed separately using analysis of covariance with age and gender as covariates of noninterest. 

Similar to our recent studies [63,64,65], a threshold of voxel-wise *p* < 0.005 uncorrected and cluster-level *p* < 0.05 false discovery rate (FDR) corrected were applied in group analysis. Because the ACC, mPFC, insula, and amygdala play crucial roles in the reward and pain modulation pathways [23,24,66], we predefined these areas as region of interests (ROIs). For ROIs (as defined by Automated Anatomical Labeling atlas), a threshold of voxelwise *p* < 0.005 was used in data analysis. To correct for multiple comparisons, Monte Carlo simulations using the 3dFWHMx and 3dClustSim [AFNI (https://afni.nimh.nih.gov)] were applied. 

To explore the association between the rsFC z-score changes and clinical outcome changes, we also extracted the average z-score values of the significantly altered rsFC clusters before and after treatment (sham versus real). We then performed a partial correlation analysis to test correlations between the significantly altered rsFC clusters and pain bothersomeness score changes, controlling for age and gender.

## 9. Results

Seventy-nine subjects with cLBP were included in this study. Fourteen patients dropped out of the study before the baseline MRI sessions, and 11 patients dropped out before the first acupuncture treatment. Fifty-four patients received four weeks of real or sham acupuncture treatment with augmented or limited context, and four patients did not finish all treatment sessions. In total, 50 patients were included in the final analysis (Figure 1). Demographic characteristics for the 50 subjects who completed all study procedures are detailed in Table 1 and Table S2. There were no significant differences between the four groups in age, gender, depression, and bothersomeness scores at baseline (all *p* > 0.05). 

## 10. Clinical Outcomes

Patients’ pain bothersomeness scores after all treatments were reduced significantly in all four groups (augmented real *p* < 0.001, augmented sham *p* = 0.033, limited real *p* = 0.001, limited sham *p* = 0.013) (Table 1). In addition, we found that real acupuncture produced significantly greater pain bothersomeness score reduction than sham acupuncture (*p* = 0.043), but there was no significant difference between the augmented and limited context groups (*p* = 0.21). 

A two-sample t-test showed no significant differences in expectation (measured by the expectations for relief scale) between the augmented context and limited context groups after treatment Session 1, Session 4, and Session 6 (Table S3). This result, along with similar bothersomeness score changes in high and low context groups, indicates that the context manipulation model in this study failed to change patients’ expectancies and modulate therapeutic effects. Thus, in the following analysis, we combined the two high-context and low-context groups to focus on the differences between real and sham acupuncture treatments.

## 11. Functional Connectivity Results

Comparisons of head movement between the real and sham acupuncture groups at baseline and after treatment, as well as before and after real or sham treatment, showed no significant differences (*p* = 0.216, *p* = 0.982, *p* = 0.698, *p* = 0.331, respectively).

**Figure 2:**
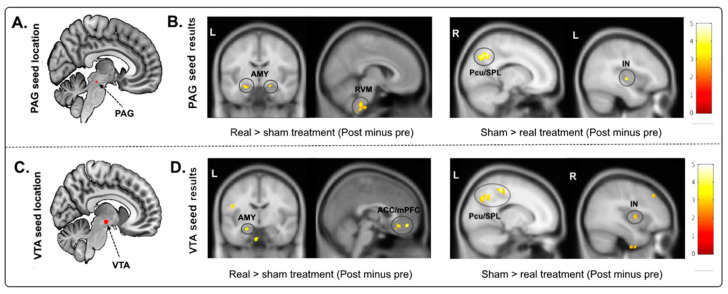
(**A**) The location of the selected PAG seed (seeds marked in red). (**B**) Compared to sham group, real group had significantly higher rsFC with the RVM and left/right amygdala and lower rsFC with the right Pcu/SPL and middle insula. (**C**) The location of the selected VTA seed. (**D**) Compared to sham group, real group had significantly higher rsFC with the bilateral ACC/mPFC and left amygdala and lower rsFC with the SPL/Pcu and right IN. Abbreviations: PAG, periaqueductal gray; rsFC, resting-state functional connectivity; RVM, rostroventromedial medulla; VTA, ventral tegmental area; Pcu/SPL, precuneus/superior parietal lobe; IN, insula; AMY, amygdala; ACC/mPFC, anterior cingulate cortex/medial frontal cortex.

The comparison of the bilateral PAG rsFC change (‘post’ minus ‘pre’) between the real and sham acupuncture treatments revealed a significant increase in rsFC between the PAG and RVM and left/right amygdala in the real acupuncture group (Table 2, Figure 2). Real acupuncture treatment produced greater rsFC decreases between the PAG and right precuneus/superior parietal lobule (Pcu/SPL) and middle insula (IN) compared to the sham group (Table 2, Figure 2).

Using the bilateral VTA as seed revealed a significantly increased rsFC change (‘post’ minus ‘pre’) with the bilateral anterior cingulate cortex/medial prefrontal cortex (ACC/mPFC) and left amygdala (Table 2, Figure 2), as well as decreased rsFC with the left superior parietal lobule/angular gyrus/precuneus (SPL/AG/Pcu) and right anterior IN in the real acupuncture group compared to the sham acupuncture group (Table 2, Figure 2). 

**Figure 3:**
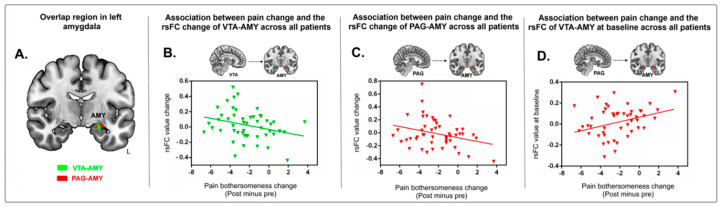
(**A**) Brain regions showed overlap at the left amygdala when using both the VTA and PAG as seeds (red, PAG seed; green, VTA seed; yellow, overlapping region). (**B**) Scatter plots indicate the correlation between change in pain bothersomeness score and change in mean *z* value of VTA-left amygdala rsFC across all cLBP subjects adjusted for age and gender. (**C**) Scatter plots indicate the correlation between change in pain bothersomeness score and mean *z* values change in PAG-left amygdala rsFC across all cLBP subjects adjusted for age and gender. (**D**) Predicting the effects of acupuncture on pain in cLBP patients with the left amygdala cluster. Correlation between prediction value (rsFC value of PAG-amygdala at baseline) and pain bothersomeness score change across all cLBP patients adjusted for age and gender. Abbreviations: VTA, ventral tegmental area; PAG, periaqueductal gray; rsFC, resting-state functional connectivity; cLBP, chronic low back pain.

Because we found an overlapping rsFC increase at the left amygdala when using both the VTA and PAG as seeds (Figure 3A), we extracted the average *z*-values of the overlapping area at the left amygdala in the two groups and performed a multiple regression analysis, including age and gender as covariates. We found a significant negative association between rsFC *z*-values at the VTA-amygdala and PAG-amygdala and corresponding pain bothersomeness score changes (*p* = 0.045, *r* = −0.30 in Figure 3B; *p* = 0.044, *r* = −0.30 in Figure 3C) across all subjects. 

To explore whether the overlapping rsFC at the left amygdala between the two treatment groups could predict treatment response, we also extracted the average *z*-values of the cluster at the PAG-left amygdala and VTA-left amygdala at baseline and applied a multiple regression analysis (controlling for age and gender) with corresponding pain bothersomeness score changes (post-treatment minus pre-treatment). We found a significant positive association between the rsFC *z*-value in the left amygdala using PAG as seed at baseline and the cLBP pain bothersomeness change (*r* = 0.30, *p* = 0.038, Figure 3D) across all subjects. This suggests that baseline PAG-AMY has the potential to predict one’s treatment response to interventions. We did not find a significant association between the rsFC z-value in the left amygdala using the right VTA as seed. 

**Figure 4:**
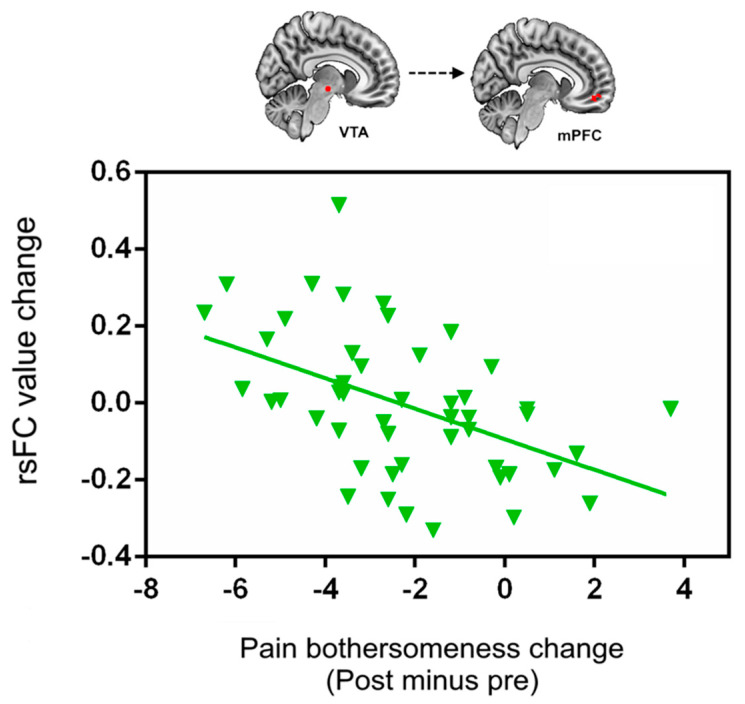
Scatter plots indicate the correlation between change in pain bothersomeness score and corresponding change in mean z value of VTA-bilateral ACC/mPFC rsFC across all cLBP subjects adjusted for age and gender. Abbreviations: VTA, ventral tegmental area; ACC/mPFC, anterior cingulate cortex/medial prefrontal cortex; rsFC, resting-state functional connectivity; cLBP, chronic low back pain.

Due to the important role of the VTA and ACC/mPFC in the reward process, we also extracted the cluster value of VTA-ACC/mPFC rsFC increases (scan 2–scan 1) and explored its association with cLBP pain changes after treatment. We found a significant association between the VTA-ACC/mPFC rsFC increases and pain bothersome score decreases (*p* < 0.001, *r* = −0.50) (Figure 4) across all subjects after treatment. 

## 12. Discussion 

We investigated the modulation effect of acupuncture on the DPMS and reward motivation systems in cLBP patients. The results showed a significant remission of pain severity in both real and sham groups after four weeks, with a greater reduction in bothersomeness score after real acupuncture. Most importantly, we found that the left amygdala was the overlapping region when using the VTA and PAG as seeds after real acupuncture compared to sham acupuncture, and the VTA/PAG-amygdala rsFC increases were associated with reduction in pain bothersomeness. Baseline PAG–amygdala rsFC can predict bothersomeness reduction after treatments. In addition, real acupuncture significantly increased PAG rsFC with the rostral ventral medulla, as well as VTA rsFC with the prefrontal cortex. Our results suggest that acupuncture may achieve treatment effects by modulating the descending pain modulation system and reward network. 

We also found that real acupuncture can produce greater improvement than sham controls. This result is consistent with findings from systematic reviews testing the efficacy of acupuncture on cLBP [7,8]. In these reviews, researchers found that although differences between real and sham acupuncture are relatively modest, acupuncture is superior to both a non-acupuncture control and sham acupuncture for the treatment of chronic pain.

Literature suggests that expectation plays an important role in the placebo response [41,67]. We did not detect significant differences between the high and low context groups as expected, and no significant expectancy score after context manipulation was detected (Table S2). In a previous study on knee osteoarthritis patients [65], we applied a conditioning-like expectancy manipulation model and found that the model was able to significantly increase the effect of acupuncture treatment for knee pain as compared to an identical acupuncture treatment without expectancy manipulation. These results may suggest that the conditioning-like model is more robust than verbal suggestion, which is consistent with findings from experimental pain studies [68]. Our findings imply that gaining patients’ trust and boosting expectancy is a complicated process, where warmness and empathy may be just two factors that can influence patients’ expectancies/beliefs. Future studies with larger sample sizes are needed to validate this finding.

The most intriguing result in our study was that rsFC of the left amygdala with the VTA and PAG was significantly increased after real acupuncture when compared to sham acupuncture. The amygdala plays a dual role of facilitating and inhibiting the modulation of pain behavior and nociceptive processing at different levels [69,70,71], and it is the core region in negative emotion processing and the DPMS [72,73]. Previous task-related fMRI studies have shown that brain activity changes at the amygdala during acupuncture needle stimulation in healthy volunteers and chronic pain patients [74,75]. Our previous study demonstrated that real acupuncture can increase rsFC between the amygdala and ACC and that this treatment is associated with clinical improvement in patients with depression [76]. Higher connectivity between the VTA/PAG and amygdala was associated with lower pain bothersomeness scores in cLBP patients. We speculate that the amygdala may be a key node that links the reward and descending pain modulation systems to produce antinociceptive effects. 

It is also important to note that the amygdala is implicated in fear-related neural circuits and has long been associated with emotional information and memory processing [73,77,78]. A number of recent reports have shown that chronic pain and a great fear of pain are intricately connected [79,80], suggesting that pain-related circuits and fear circuits may undergo some of the same neural alterations during pain processing. Hence, we speculate that changes in rsFC of PAG–AMY are also associated with the conversion of pain-induced fear associations into long-lasting memories. 

We found a significant positive association between PAG–AMY rsFC at baseline and pain bothersomeness score reduction. Studies have shown that PAG–AMY rsFC is correlated with pain ratings in cLBP patients [22,81], migraineurs [82], and healthy subjects [83]. Anatomically, the amygdala sends heavy and broad projections to the rostral midbrain, including the PAG [84], and it is a key substrate facilitating stress-conditioned analgesia, most of which appears to depend on the release of endogenous opioids [85,86]. Our results suggest that baseline PAG–AMY rsFC may be able to predict the magnitude of acupuncture treatment effects. 

We also found decreased rsFC in the middle/posterior insula and precuneus with the VTA and PAG after real acupuncture compared to sham acupuncture. The middle/posterior insula has been linked to perception of pain and possibly the regulation of chronic pain states in humans [87,88]. The precuneus is the key node of the posterior default mode network (DMN), and its rsFC is enhanced in chronic pain and associated with pain rumination [89]. Acupuncture could reduce VTA/PAG rsFC with the insula and precuneus, indicating that acupuncture may target both the DPMS and ascending pain perceptional system. 

The PAG receives and projects to broader cortical and subcortical regions, including the mPFC, ACC, precentral, postcentral, amygdala, insula, and RVM [90,91,92]. In a previous study, we found abnormal PAG rsFC with the insula, amygdala, and ACC in cLBP patients [22]. In a more recent study, we found that chronic low back pain patients showed increased low-frequency oscillations in the insula, amygdala, hippocampal/parahippocampal gyrus, and thalamus when their spontaneous low back pain intensity increased after a pain-exacerbating maneuver [93]. In this study, we found that acupuncture decreased rsFC between the PAG and RVM, another important region in the descending pain modulation system, compared to sham acupuncture in cLBP. This modulation effect of acupuncture treatment on the DPMS is consistent with our previous findings on migraine patients [60,94]. 

The VTA plays an important role in the brain’s encoding of pain processing and pain relief [26,95]. Relief of pain is rewarding [26,96,97]. Dennis and Melzack demonstrated that dopaminergic agents improve symptoms of pain and promote analgesia [98]. Studies have also shown that the VTA sends projections to the nucleus accumbens (NCs), limbic system (e.g., hippocampus and amygdala), and prefrontal cortex (e.g., mPFC, ACC, and OFC), forming the mesocorticolimbic system [27,28,29,30,31]. 

We found that VTA rsFC was significantly increased at the mPFC/ACC after real acupuncture compared to sham acupuncture. This result is consistent with previous studies showing that the reward pathway plays an important role in acupuncture treatment [65,99,100]. The mPFC/ACC, a key region of the DMN, is an important brain region dedicated to representing the hedonic properties of reward, focusing on learning appropriate action-reward contingencies and selecting those actions that potentially lead to a reward [101,102]. Studies show that the opioid-rich rACC/mPFC [103] is involved in the self-regulation of pain, such as placebo analgesia [104,105] and treatment of chronic pain [60,65,106]. In a recent study, we found that patients with chronic low back pain are associated with decreased VTA rsFC with the rACC and MPFC compared to pain-free controls [32]. In this study, we found that acupuncture can increase VTA-mPFC/ACC connectivity, and this increase was associated with acupuncture-induced improvement. Taken together, these findings demonstrate the important role of the mesocorticolimbic reward pathway in acupuncture treatment of chronic low back pain. 

Several limitations of this study should be mentioned. First, the current study was conducted over a four-week period. Therefore, the effects observed in the fMRI only represent short- to mid-term influences. Second, the sample size of the study was relatively small. Thus, our results should be verified by studies with larger sample sizes. Finally, only resting-state MRI scans were applied at baseline and after the six treatments. We did not include task-related fMRI (scans during needle stimulation) due to the fact that (1) the acupoints selected in this study were mainly on the back, where it is difficult to manipulate the needle during an MRI scan; and (2) a previous study indicated that a standard acupuncture needle may reduce MRI signal quality [107]. We therefore believe that our result is not an acute effect of needle stimulation, but rather a long-term effect of multiple treatments. 

In summary, we found that real acupuncture analgesia was associated with increased VTA/PAG—amygdala functional connectivity compared to sham acupuncture, indicating that the amygdala may be a key node that links the descending pain modulation system and reward system to produce antinociceptive effects. We were also able to use baseline PAG—amygdala functional connectivity to predict the four-week treatment response. The unique characteristic of simultaneously modulating resting state functional connectivity of key regions in the descending pain modulation system and reward network highlights the potential of acupuncture in the management of chronic pain. 

## Figures and Tables

**Table 1:** Demographic and clinical information of each group.

Item	Real Acupuncture		Sham Acupuncture		Real vs. Sham		Augmented vs. Limited	
	Augmented Real (12)	Limited Real (12)	Augmented Sham (13)	Limited Sham (13)	*T/X^2^*	*p*	*T/X^2^*	*p*
Age	43.00 (11.09)	34.98 (13.16)	40.02 (13.51)	39.51 (14.40)	−2.71	0.787	1.05	0.225
Female/male	8/4	8/4	8/5	7/6	0.43	0.514 ^†^	0.09	0.771
Beck Depression Inventory	6.66 (6.05)	12.18 (11.43) ^#^	6.69 (5.58)	6.61 (5.09)	1.41	0.16	−1.21	0.23
Baseline pain bothersomeness	5.97 (1.60)	6.23 (1.81)	5.22 (1.78)	5.33 (1.69)	1.73	0.091	−0.39	0.711
Post-treatment pain bothersomeness	3.59 (2.16)	3.03 (2.59)	3.60 (2.47)	3.51 (2.51)	0.37	0.715	0.47	0.692
Change in pain bothersomeness	−2.38 (1.45)	−3.21 (2.45)	−1.62 (2.41)	−1.81 (2.26)	−1.75	0.043 *	0.81	0.210 *

Notes: ^†^, the *p* value was obtained by chi-square test; other *p* values were obtained by a two-sample *t*-test; *, the *p* value was obtained by a one-side analysis; ^#^, n = 11 in the limited real group.

**Table 2:** Significantly different brain regions identified after real and sham treatments in patients with chronic low back pain (cLBP), including age and gender as covariates.

Seed	Contrast	Brain Regions	Cluster Size (Voxels)	MNI Coordinates (*x*, *y*, *z*)	Peak *z*-Value
VTA	Real > sham (post minus pre)	Bilateral ACC/mPFC *	118	−2, 28, −20	4.38
		Bilateral mPFC *	51	2, 48, −12	4.09
		Left amygdala *	19	−24, −8, −18	3.25
	Sham > real (post minus pre)	Left SPL/AG	119	−22, −66, 62	4.84
		Left precuneus/SPL	156	−8, −56, 64	3.71
		Left SPL/AG	134	−30, −50, 50	4.58
		Right anterior insula *	39	38, 0, 6	3.49
PAG	Real > sham (post minus pre)	RVM *	83	6, −36, −48	3.82
		Right amygdala *	33	20, −16, −14	3.72
		Left amygdala *	28	−24, −10, −18	3.72
	Sham > real (post minus pre)	Right precuneus/SPL	190	14, −66, 40	4.19
		Left insula *	23	−38, −14, 0	4.06

Notes: *, results were significant at cluster *p* < 0.05 after 3dFWHMx and 3dClustSim correction. Other results were significant at cluster *p* FDR < 0.05 corrected at the whole brain level. Abbreviations: VTA, ventral tegmental area; PAG, periaqueductal gray; ACC/mPFC, anterior cingulate cortex/medial frontal cortex; SPL/AG, superior parietal lobe/angular gyrus; RVM, rostroventromedial medulla.

## Supplementary Materials

The following are available online at https://www.mdpi.com/2077-0383/9/6/1719/s1, Table S1: Acupoint descriptions for acupuncture and sham acupuncture treatment; Table S2: Medication usage at baseline in real and sham groups; Table S3: ERS for different groups at different sessions.
