# Supplementary material for: Acupuncture Treatment Modulates the Connectivity of Key Regions of the Descending Pain Modulation and Reward Systems in Patients with Chronic Low Back Pain
Source: J Clin Med. 2020 Jun 3;9(6):1719. doi: 10.3390/jcm9061719 (PMC7356178; doi:10.3390/jcm9061719)
Supplement: Supplementary file 1 [file jcm-09-01719-s001.pdf]

Supplementary Materials

High and Low Context Manipulation

The discussion included topics about 1) LBP symptoms, 2) other medical symptoms, 3) psychosocial history, Chinese medicine intake, and how cLBP has affected the patient's relationships and lifestyle, and 4) how well the patient understands the "cause" and "meaning" of their condition. The acupuncturist incorporated five primary behaviors: 1) exuding a warm, friendly manner, 2) active listening, 3) empathy, 4) 20 seconds of thoughtful silence while feeling the pulse or pondering the treatment plan, and 5) communication of confidence and positive expectation.

After the initial interaction, the acupuncturist performed either real or sham acupuncture treatment. At the end of the treatment, the practitioner removed the needles and provided words of encouragement and a positive, supportive expectancy environment in subsequent treatments without 30 minutes of interaction.

The quality and duration of the acupuncture stimulation/needling for both "augmented" and "limited" context groups were identical in all aspects. Subsequent treatments for the "limited" context group were conducted in a neutral, business-like manner.

The 5 treatment sessions following the initial session continued the real/sham acupuncture treatments and maintained the respective "augmented" or "limited" context that began in treatment session 1.

All participants were allowed to continue their existing medication. Medication use per self-report was limited to non-steroidal anti-inflammatory drugs (NSAIDs, e.g., ibuprofen, Motrin, Advil, and Naproxen) and acetaminophen (e.g., Tylenol). Additional non-pharmacological methods of self-reported pain management included chiropractic massages, physical therapy, and exercises.

To assess fidelity to the contextual treatment protocol, we videotaped all treatment sessions. However, subjects had the option to decline being recorded.

Table 1. Acupoint descriptions for acupuncture and sham acupuncture treatment (1 cun = ~2cm; a measurement relative to the body dimensions of each patient, defined by conventional TCM guidelines).

Acupoints for cLBP [7 regular acupoints plus 2 to 6 ashi (tender) points]			
Du 3:	In the depression below the spinous process of the 4th lumbar vertebra.	BL23:	1.5 cun lateral to the lower border of the spinous process of the second lumbar vertebra.
BL 40:	Midpoint of the transverse crease of the popliteal crease.	KD-3:	Depression between the medial malleolus and the tendocalcaneus level with the vertex of the medial malleolus.
Sham Points for cLBP (12 total)			
SH-1-8:	About 5 cun lateral from the lateral bladder meridian on both sides (bilateral with each side 4 points) and level with BL23-26 *	SH-9-10:	Two non-acupuncture points on the thigh: about 3 cun medially and 3 cun caudally from BL 37 (bilateral)
SH-11-12:	About 1 cun inferior and 1 cun medial to BL57 (bilateral)**		

*This would make the locations level with just below L1, L2, L4, and L5.

Table 2. Medication usage at baseline in real and sham groups.

Medication usage	Real acupuncture	Sham acupuncture
Non-opioid analgesics	6	4
Benzodiazepines	3	2
Muscle relaxant	3	6
No medication	12	14
Chi-square = 1.67, p = 0.357		

Table 3. ERS for different groups at different sessions.

Group	n	Expectancy of treatment			
		Baseline	Session 1	Session 4	Session 6
Augmented context	25	7.0±2.1	7.2±2.8	7.0±2.6	7.0±3.0
Limited context	25	5.3±2.9	6.3±2.7	6.2±2.9	6.1±3.0
Real acupuncture	24	6.4±2.9	7.6±2.4	7.4±2.6	7.1±2.6
Sham acupuncture	26	6.0±2.5	6.0±2.9	6.0±2.8	6.1±3.3
